# Sympathetic Activation Promotes Sodium Glucose Co-Transporter-1 Protein Expression in Rodent Skeletal Muscle

**DOI:** 10.3390/biomedicines12071456

**Published:** 2024-07-01

**Authors:** Jennifer R. Matthews, Lakshini Y. Herat, Markus P. Schlaich, Vance B. Matthews

**Affiliations:** 1 Dobney Hypertension Centre, School of Biomedical Sciences—Royal Perth Hospital Unit, University of Western Australia, and Royal Perth Hospital Research Foundation, Crawley, WA 6000, Australia; jen.matthews@uwa.edu.au (J.R.M.); lakshini.weerasekera@uwa.edu.au (L.Y.H.); 2 Dobney Hypertension Centre, School of Medicine—Royal Perth Hospital Unit, University of Western Australia, Perth, WA 6000, Australia; markus.schlaich@uwa.edu.au; 3 Departments of Cardiology and Nephrology, Royal Perth Hospital, Perth, WA 6000, Australia; 4 Neurovascular Hypertension & Kidney Disease Laboratory, Baker Heart and Diabetes Institute, Melbourne, VIC 3004, Australia

**Keywords:** diabetes, skeletal muscle, sglt1, sglt2, hypertension, blood pressure, glucose, sodium

## Abstract

The hyperactivation of the sympathetic nervous system (SNS) is linked to obesity, hypertension, and type 2 diabetes, which are characterized by elevated norepinephrine (NE) levels. Previous research has shown increased sodium-dependent glucose cotransporter 1 (SGLT1) protein levels in kidneys of hypertensive rodents, prompting investigation into the expression of SGLT1 in various tissues, such as skeletal muscle. This study aimed to assess (i) whether skeletal muscle cells and tissue express SGLT1 and SGLT2 proteins; (ii) if NE increases SGLT1 levels in skeletal muscle cells, and (iii) whether the skeletal muscle of neurogenically hypertensive mice exhibits increased SGLT1 expression. We found that (i) skeletal muscle cells and tissue are a novel source of the SGLT2 protein and that (ii) NE significantly elevated SGLT1 levels in skeletal muscle cells. As SGLT2 inhibition (SGLT2i) with Empagliflozin increased SGLT1 levels, in vivo studies with the dual inhibitor SGLT1/2i, Sotagliflozin were warranted. The treatment of neurogenically hypertensive mice using Sotagliflozin significantly reduced blood pressure. Our findings suggest that SNS activity upregulates the therapeutic target, SGLT1, in skeletal muscle, potentially worsening cardiometabolic control. As clinical trial data suggest cardiorenal benefits from SGLT2i, future studies should aim to utilize SGLT1i by itself, which may offer a therapeutic strategy for conditions with heightened SNS activity, such as hypertension, diabetes, and obesity.

## 1. Introduction

Skeletal muscle is the most abundant muscle found in the body. It has a multitude of functions, including inducing movement, stabilizing joints, and sustaining body posture and position [1]. While skeletal muscle is primarily responsible for converting chemical energy into mechanical energy, it is also important for producing body heat by contracting muscles and promoting shivering in cases of extreme cold. Additionally, these muscles serve as the storage site for carbohydrates and amino acids, therefore contributing to basal energy metabolism [1].

While the skeletal muscle performs many functions, as discussed above, one of the other most significant functions is that of glucose uptake. Glucose is the major source of energy for the majority of tissues, and skeletal muscle is responsible for 80–90% of insulin-stimulated glucose uptake in the body, particularly via glucose transporter 4 (GLUT4) [2]. More recently identified glucose and sodium co-transporters are sodium glucose co-transporters (SGLT) 1 and 2.

It has been shown that SGLT1 is not only expressed in the small intestine and the heart, but also in skeletal muscle [3], and, more recently, to be widely expressed in the retina [4]. Unlike SGLT1, SGLT2 is particularly limited to the proximal tubules of the kidneys and areas of the eye such as the retina [5], cornea [6], and lens [7]. On the other hand, one study has demonstrated that the gene for *Sglt2* (*slc5a2*) is expressed at the mRNA level in skeletal muscle; however, protein expression was not detected [3]. Future studies are warranted to ascertain if skeletal muscle is indeed a source of the SGLT2 protein.

Studies addressing the clinical impact of SGLT1 and SGLT2 inhibition (SGLT1/2i) provide critical insight into the importance of the regulation of these two members of the SGLT family. Inhibitors of SGLT2 were developed based on the anti-diabetic action initiated by inhibiting renal glucose reabsorption, thereby increasing glucosuria. Of substantial clinical importance, extensive clinical trials involving various SGLT2 inhibitors (SGLT2i) have exhibited both cardiorenal and metabolic benefits [8] through several novel mechanisms, including sympathoinhibition, as shown by our team [9]. A critical question currently under investigation is whether using a dual SGLT1/2 inhibitor, such as Sotagliflozin (SOTAG), may offer greater therapeutic benefits compared to single SGLT2 inhibitors, such as Empagliflozin (EMPA), in the treatment of cardiorenal disease in both patients with and without diabetes. As dual SGLT1/2 inhibitors represent a relatively novel pharmacological intervention compared to monotherapy with SGLT2 inhibitors, the scope of research findings pertaining to SGLT1/2 inhibitors is currently limited. Highlighting the importance of SOTAG-related studies, two clinical trials utilizing SOTAG showed that in patients with type 2 diabetes (T2D) and Chronic Kidney Disease (CKD), the administration of SOTAG significantly reduced the rate of hospitalization and urgent care visits for heart failure compared to a placebo [10,11].

Furthermore, it seems significantly relevant to examine the mechanisms that regulate glucose and sodium transport in skeletal muscle. This is important because, for example, in sarcopenia, there is a loss of skeletal muscle mass and strength, and both hyperglycemia (high glucose levels) and high salt intake have been associated with increased risk of sarcopenia [12,13]. While hyperglycemia negatively affects the regenerative capability of the skeletal muscle satellite cells [12], high salt intake may also contribute to fat accumulation resulting from leptin resistance [14] and promotes the muscle weakness associated with sarcopenia [13]. Thus, in conditions such as diabetes-induced sarcopenia, inhibiting excessive sodium and glucose uptake in muscle is clinically relevant.

Given that *Sglt2* has only been shown to be expressed at the mRNA level in skeletal muscle, our study was particularly novel, as it aimed to determine if the SGLT2 protein exists within skeletal muscle. In addition, our study fills a gap in the literature, as there are limited studies on the regulation of SGLT1, especially in the context of SGLT2 inhibition (SGLT2i) therapy and how it interacts with the sympathetic nervous system (SNS). In our current study, we investigate the mechanisms underlying SGLT1 regulation in skeletal muscle.

This study aims to demonstrate that either selective SGLT1 inhibition (SGLT1i) or dual SGLT1/2 inhibition (SGLT1/2i) could be beneficial in treating diseases associated with SNS activation, such as diabetes and hypertension, and that skeletal muscle may be a target organ for these therapies. We hypothesize that both SGLT2 inhibition and sympathetic nervous system (SNS) activation may upregulate SGLT1 expression.

## 2. Materials and Methods

### 2.1. L6 Cell Culture

L6 rodent skeletal muscle cells were cultured in growth medium containing 1 g/L low-glucose Dulbecco’s Modified Eagle Medium (LG-DMEM) containing L-glutamine (1%), streptomycin (2%) and fetal calf serum (FCS) (10%), (Thermo Fisher, Melbourne, Australia) in a 75 cm^2^ flask until 70% confluency was achieved. At this point, the cells underwent trypsinization to allow for detachment from the flask to take place for their transfer into a Corning Cell Bind 6-well plate (Corning, NY, USA) in 2 mL of growth medium.

The differentiation process started once the cells achieved 70% confluency in the growth medium. The differentiation medium contained low-glucose Dulbecco’s Modified Eagle Medium (LG-DMEM) containing L-glutamine (1%), streptomycin (2%), and fetal calf serum (FCS) (2%). The differentiation medium was changed every two days for seven days [15]. At this point, the wells were treated appropriately, as indicated below.

### 2.2. Treatment with Norepinephrine (NE)

Our team has previously published the optimal NE treatment conditions for SGLT1 and SGLT2 protein studies in HK2 cells [16]. Differentiated L6 skeletal muscle cells were treated with either the vehicle (VEH, Baxter water) or 10 µM norepinephrine (NE, Sigma-Aldrich, Sydney, Australia) in 2 mL of low-glucose DMEM growth medium for a duration of 48 h. The norepinephrine was protected from light during both its preparation and the experimental procedures.

### 2.3. Sole SGLT2 Inhibitors and Dual SGLT1/2 Inhibitor Treatment

Based on the previously published literature, the SGLT2i Empagliflozin (EMPA; Ark Pharma Scientific Limited, Wuhan, China) was used at 30 µM for in vitro purposes [17,18]. Stock solutions (0.03 M) were prepared and diluted to a ratio of 1 in 1000 in low-glucose differentiation media. Dimethyl sulphoxide (DMSO) was used to dissolve the SGLT2i, and the control cells were treated with equivalent volumes of DMSO (the vehicle) compared to the SGLT2i treatment. Incubation times were chosen based on previously published data [17]. To allow for comparability, SOTAG was also prepared as a 0.03 M stock solution. The stock solution was diluted at a 1 in 1000 dilution in low-glucose differentiation media.

### 2.4. Collection of Protein Cell Lysates

After cells were treated, they were gently placed on ice, where the medium was removed and discarded. This was followed by two 1× cold PBS washes of each well (2 mL per well). The monolayer was then lysed with 60 µL of lysis buffer containing 1× phosphatase inhibitor, 1× protease inhibitor, and a cytosolic extraction buffer (CEB). The CEB consisted of 10 mM 4-(2-hydroxyethyl)-1-piperazineethanesulfonic acid (HEPES), pH 7.5, 14 mM KCl, 5% glycerol, and 0.2% nonidet P-40 (Igepal). After adding the lysis buffer to cells, adhered L6 cells were then scraped and transferred to a 1.5 mL Eppendorf tube to be stored at −80 °C for 24 h. The cell lysates were thawed and centrifuged at 12,000 rpm at 4 °C for 10 min. Supernatants were gently transferred to a new 1.5 mL Eppendorf tube for protein quantitation.

### 2.5. Protein Quantification Using the Bradford Assay

Sample protein concentrations were determined using the Bradford assay. The bovine serum albumin (BSA) protein standards were added to 96-well plates in duplicate with concentrations ranging from 0 to 6 µg/µL. In separate wells, 1 µL of protein sample was added in duplicate to the same 96-well plate. Then, 250 µL of the Bradford protein assay reagent was prepared for each sample well (50 µL of Bio-Rad dye reagent concentrate [Bio-Rad Laboratories, South Granville, NSW, Australia] and 200 µL of Milli-Q water) and added to each well. The absorbance of samples was then determined after reading the plate at 595 nm, which gave rise to the development of a standard curve (OD versus BSA protein standard concentrations). Unknown protein sample concentrations were then calculated using the standard curve. The samples were then prepared (10 µg/100 µL) for SGLT1 determination.

### 2.6. Enzyme-Linked Immunosorbent Assay (ELISA)

L6 lysates were analyzed for SGLT1 protein expression levels using a commercially available rodent SGLT1-specific ELISA kit (Cloud clone—SEE 381Mu (Wuhan, China)) as per the manufacturer’s instructions.

### 2.7. SGLT1 and SGLT2 Immunocytochemistry of L6 Cells

SGLT1 and SGLT2 proteins were found in L6 cells utilizing the immunocytochemistry technique. The cells were fixed in a methanol/acetone solution (1:1) on a 6-well plate, and endogenous peroxidases were blocked using 3% hydrogen peroxide (H_2_O_2_). This step was followed by blocking with 10% fetal calf serum (FCS) in Triton X/phosphate-buffered saline (PBS). The primary antibodies, (i) anti-SGLT1 (1:500, Rabbit antibody, Novus, NBP2.20338, Centennial, CO, USA) and (ii) anti-SGLT2 (1:500, Rabbit antibody, Santa Cruz Biotechnology, sc98975, Sydney, Australia), were diluted in 0.05% Triton X/PBS, added to each well, and incubated overnight at 4 °C. The following day, a horseradish peroxidase-conjugated anti-rabbit secondary antibody (1:100, Santa Cruz Biotechnology, Sydney, Australia) was added to each well. The cells were washed, and the expressions of SGLT1 and SGLT2 were detected using diaminobenzidine (DAB) prior to visualization with a high-powered microscope.

### 2.8. Animal Work

Twelve-week-old BPN/3J (blood pressure normal) and BPH/2J (blood pressure high) male mice were obtained for experiments from the Ozgene Animal Resource Centre (Ozgene ARC, Perth, Australia). The BPH/2J Schlager mouse possesses neurogenic hypertension [19,20] and serves as a highly relevant model for human disease. They exhibit increased sympathetic activity [21], heightened heart rate, and elevated blood pressure [21,22], all driven by neurogenic mechanisms [23]. All animal experiments were carried out at the Harry Perkins Institute for Medical Research animal holding facility (Perth, WA, Australia). Animal ethics were approved by the Harry Perkins Institute for Medical Research Animal Ethics Committee (AE284).

The mice were housed individually under a 12 h light/dark cycle at 21 ± 2 °C and were provided with a standard chow diet (Specialty Feeds, Glen Forrest, WA, Australia) with access to food and drinking water ad libitum. Following a 7-day acclimatization period, either the dual SGLT1/2 inhibitor (SOTAG; Med Chem Express, Princeton, NJ, USA; 25 mg/kg/day) or the vehicle (dimethylsulfoxide [DMSO]) was administered to each mouse via drinking water for a period of 2 weeks. Drinking water containing the inhibitor or vehicle was freshly prepared and replenished weekly. The blood pressure measurements were conducted on 13-, 14-, and 15-week-old BPH/2J mice as per our previous studies [21], using a non-invasive computer-automated multichannel blood pressure analysis system MC4000 (Hatteras Instruments, Cary, NC, USA). Quadricep skeletal muscle tissue was dissected from our 15-week-old BPN/3J or BPH/2J (Schlager) mice [21,24], fixed in paraformaldehyde, and embedded in wax, following the protocol described by Herat et al., 2020 [24].

### 2.9. SGLT1 Immunohistochemistry of Quadricep Skeletal Muscle

We conducted SGLT1 immunohistochemistry on the quadricep muscle of BPN/3J mice and BPH/2J mice. Quadricep muscle from both BPN/3J and BPH/2J mice were sectioned (5 µm) onto positively charged microscope slides, deparaffinized in xylene, and rehydrated in ethanol. Antigen retrieval was performed by heating slides in an EDTA buffer (pH 8.5; Sigma-Aldrich, Sydney, NSW, Australia). After antigen retrieval, the slides were washed in PBS/0.1% Tween (2 × 5 min). Tissue sections were outlined with a paraffin pen, blocked with 3% H_2_O_2_ (10 min), washed with PBS/0.1% Tween (2 × 5 min), and then blocked with 5% FCS in PBS/0.1% Tween in a humidified chamber (1 h). Subsequently, the sections were incubated overnight with primary anti-SGLT1 antibodies at 4 °C in a humidified chamber. A novel immunohistochemical detection method was employed using a combination of primary antibodies from Abcam (Rabbit anti-SGLT1, 14685, IgG, Polyclonal, 1:180) and Novus Biologicals (Rabbit anti-SGLT1, NBP2.20338, IgG, Polyclonal, 1:100) [25]. After overnight incubation, the sections were washed with PBS/0.1% Tween (3 × 5 min) and incubated with HRP-conjugated anti-rabbit secondary antibody (1:100, Santa Cruz Biotechnology, Sydney, Australia) in PBS/0.1% Tween (1 h). This was followed by incubation with diaminobenzidine (DAB). Finally, the tissue sections were dehydrated and mounted with DPX mounting media (Sigma-Aldrich, Sydney, Australia).

### 2.10. SGLT2 Immunohistochemistry of Quadricep Skeletal Muscle

We conducted SGLT2 immunohistochemistry on the quadricep muscle of BPN/3J mice and BPH/2J mice. Quadriceps from both BPN/3J and BPH/2J mice were sectioned (5 µm) onto positively charged microscope slides and deparaffinized in xylene, followed by rehydration in ethanol. Antigen retrieval was achieved by heating the slides in the EDTA buffer (pH 8.5; Sigma-Aldrich, Sydney, NSW, Australia). Following antigen retrieval, the slides were washed in PBS/0.1% Tween (2 × 5 min). The tissue sections were outlined with a paraffin pen, blocked with 3% H_2_O_2_ (10 min), washed with PBS/0.1% Tween (2 × 5 min), and then blocked with 5% FCS in PBS/0.1% Tween in a humidified chamber (1 h).

Subsequently, the sections were incubated overnight with primary anti-SGLT2 antibodies at 4 °C in a humidified chamber. A novel immunohistochemical detection method was employed using a combination of primary antibodies from Santa Cruz Biotechnology (Mouse anti-SGLT2, sc393350, 1:50) and Novus Biologicals (Rabbit anti-SGLT2, NBPI-92384, 1:200). After overnight incubation, the sections were washed with PBS/0.1% Tween (3 × 5 min) and incubated with both HRP-conjugated anti-rabbit (1:100, Santa Cruz Biotechnology, Sydney, Australia) and anti-mouse (1:100, Santa Cruz Biotechnology, Sydney, Australia) secondary antibodies in PBS/0.1% Tween for 1 h. This was followed by incubation with diaminobenzidine (DAB). Finally, the slides were dehydrated and mounted with DPX mounting media (Sigma-Aldrich, Sydney, Australia).

### 2.11. Tissue Imaging and Analysis

Images were obtained using the inverted microscopy system Nikon Eclipse Ti (Nikon, Tokyo, Japan) equipped with a CoolSNAP HQ2 digital camera (Photometrics, Tucson, AZ, USA), connected to a computer running NIS-Elements Advanced Research image analysis software (Nikon, Tokyo, Japan; https://www.microscope.healthcare.nikon.com). The mean SGLT1 staining expression intensity scores were calculated on a scale of 0–3 (0 = absent expression; 1 = low expression; 2 = intermediate expression; and 3 = high expression). Expression scores were determined by two independent scorers to ensure unbiased results were produced.

### 2.12. Statistical Analysis

All normally distributed data were analyzed using the two-tailed Student’s *t*-test or one-way ANOVA, and *p* ≤ 0.05 was considered as a significant threshold. Normality Shapiro–Wilk and Kolmogorov–Smirnov testing and two-tailed Student’s *t*-tests were conducted using GraphPad Prism 10 software (San Diego, CA, USA). One-way ANOVAs were conducted using JAMOVI 2.0 software.

## 3. Results

### 3.1. Skeletal Muscle Is a Novel Source of SGLT2 Protein

#### 3.1.1. Immunocytochemistry Staining of Differentiated L6 Skeletal Muscle Cells Shows SGLT2 Protein Expression

**Figure 1:**
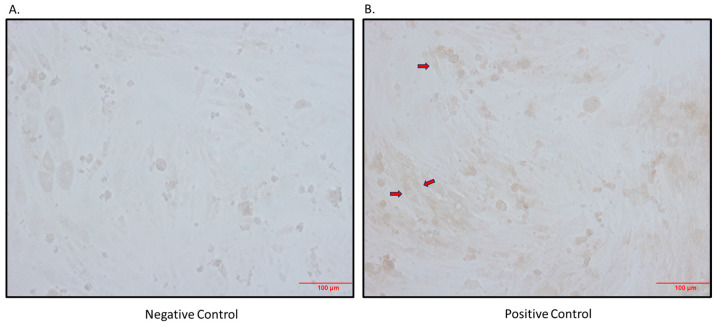
Immunocytochemistry detection of SGLT2 protein in differentiated L6 cells. (**A**) Negative control (primary antibody omitted); (**B**) positive control (primary antibody added; arrows indicate brown staining); magnification 200×; scale bars: 100 μm.

It has previously been shown that *Sglt2* mRNA is expressed in rodent skeletal muscle [3]; however, it has not yet been shown to be expressed at the protein level. Further support for SGLT2 being possibly expressed at the protein level in skeletal muscle is the fact that the SGLT2i, Canagliflozin, was used in the L6 skeletal muscle cell line [26]. Utilizing the L6 cell line, we found that the SGLT2 protein is indeed expressed in differentiated L6 cells (Figure 1).

#### 3.1.2. Skeletal Muscle Expresses SGLT2 In Vivo

**Figure 2:**
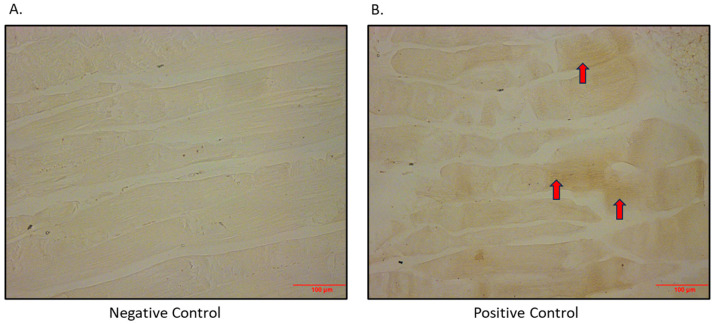
Immunohistochemical detection of SGLT2 in quadricep skeletal muscle of BPH/2J mice. (**A**) Negative control (primary antibody omitted); (**B**) positive control (primary antibody added; arrows indicate brown staining). Magnification 200×; scale bar: 100 μm.

After determining that the SGLT2 protein is expressed in the L6 skeletal muscle cell line, we also set out to show that this is an in vivo phenomenon. The SGLT2 protein was shown to be expressed heterogeneously in quadricep skeletal muscle from BPH/2J mice (Figure 2).

### 3.2. Skeletal Muscle Is a Novel Source of SGLT1 Protein

#### 3.2.1. Immunocytochemistry Staining of Differentiated L6 Skeletal Muscle Cells and Quadricep Muscle Shows SGLT1 Protein Expression

**Figure 3:**
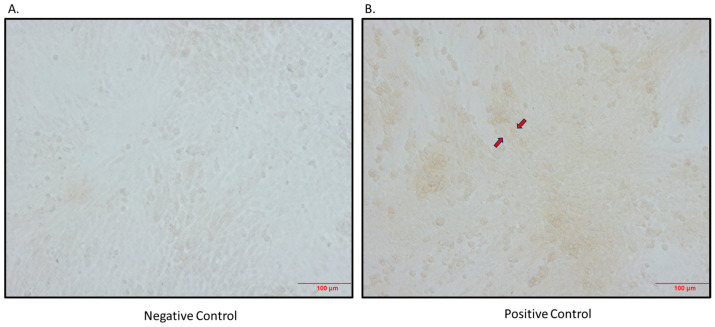
Immunocytochemical detection of SGLT1 in differentiated L6 skeletal muscle cells. (**A**) Negative control (primary antibody omitted); (**B**) positive control (primary antibody added; arrows indicate brown staining); magnification 200×; scale bar: 100 μm.

**Figure 4:**
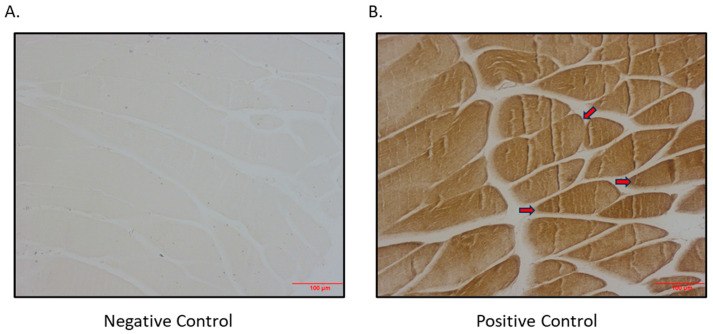
Immunohistochemical detection of SGLT1 expression in quadricep skeletal muscle of BPH/2J mice. (**A**) Negative control (primary antibody omitted); (**B**) positive control (primary antibody added; arrows indicate brown staining); magnification 200×; scale bar: 100 μm.

As SGLT2 is expressed in skeletal muscle, we aimed to determine if there was also SGLT1 expression in skeletal muscle. We indeed showed that SGLT1 is expressed in differentiated L6 skeletal muscle cells (Figure 3) and also in quadricep skeletal muscles of mice (Figure 4).

#### 3.2.2. SGLT2 Inhibition (SGLT2i) Promotes SGLT1 Expression in Differentiated L6 Skeletal Muscle Cells

**Figure 5:**
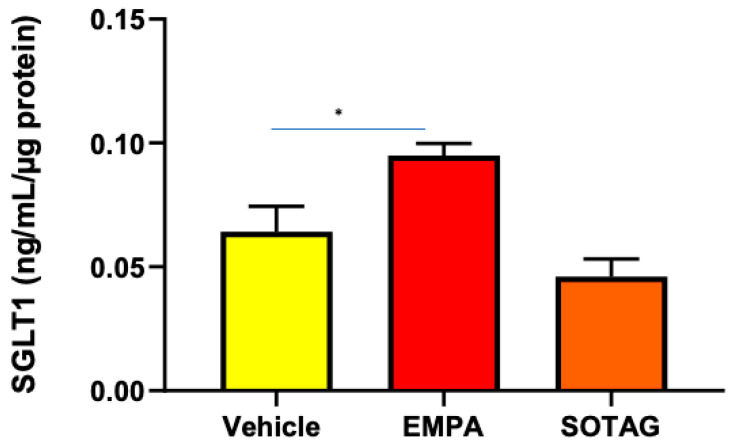
SGLT1 protein levels are elevated in differentiated L6 skeletal cells following SGLT2i but not combined SGLT1/2i. Cells were treated with vehicle, EMPA, or SOTAG after 7 days of differentiation; data represented as means ± SEM; n = 4/group; statistical analysis was conducted using one-way ANOVA; * *p* = 0.049.

In previous studies, we have shown that SGLT2i produces a compensatory increase in SGLT1 expression in the eye and the kidney [4,25]. Therefore, we wanted to ascertain if SGLT2i promotes the same phenomenon in skeletal muscle. We discovered that SGLT2i promoted SGLT1 expression in differentiated L6 skeletal muscle cells, while the combined SGLT1/2i, Sotagliflozin, did not (Figure 5). The SGLT2i-mediated elevation of SGLT1 warrants the utilization of an SGLT1/2i such as Sotagliflozin to inhibit the activity of both SGLT1 and SGLT2. Interestingly, SGLT2 protein levels in differentiated L6 cells were not differentially regulated by SGLT2i or combined SGLT1/2i (Supplementary Figure S1).

#### 3.2.3. The Primary Neurotransmitter of the SNS, NE, Significantly Increases SGLT1 Levels in Differentiated L6 Skeletal Muscle Cells

**Figure 6:**
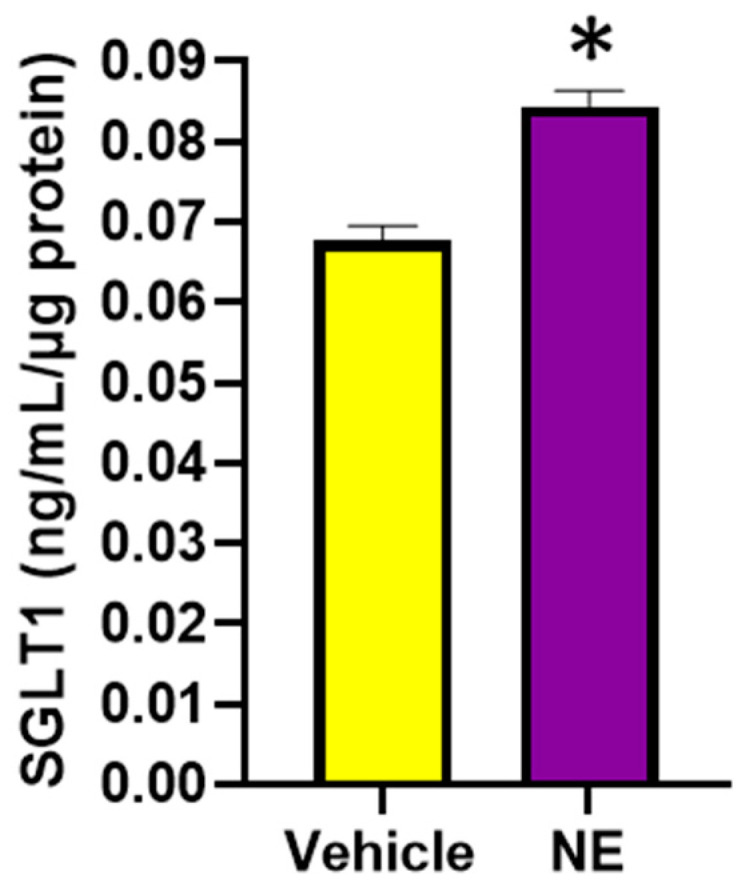
SGLT1 protein levels are significantly elevated following the NE (10μM) treatment of differentiated L6 cells. SGLT1 levels were measured in whole-cell lysates, 48 h post treatment; data represented as means ± SEM; n = 3–5/group; statistical analysis was conducted by two-tailed Student’s *t*-tests; * *p* < 0.001.

We were interested in discovering the mechanisms of SGLT1 regulation, as SGLT1 is a therapeutic target in metabolic syndrome. Therefore, we aimed to determine whether norepinephrine (NE), the primary neurotransmitter of the sympathetic nervous system (SNS), which is elevated in conditions such as hypertension, diabetes, and obesity, may upregulate SGLT1 expression (Figure 6). Indeed, we were able to demonstrate that this neurotransmitter does in fact significantly promote SGLT1 protein levels in the differentiated L6 skeletal muscle cells.

#### 3.2.4. Norepinephrine (NE) Induces an Increase in Interleukin-6 (IL-6) Levels in Differentiated L6 Cells

**Figure 7:**
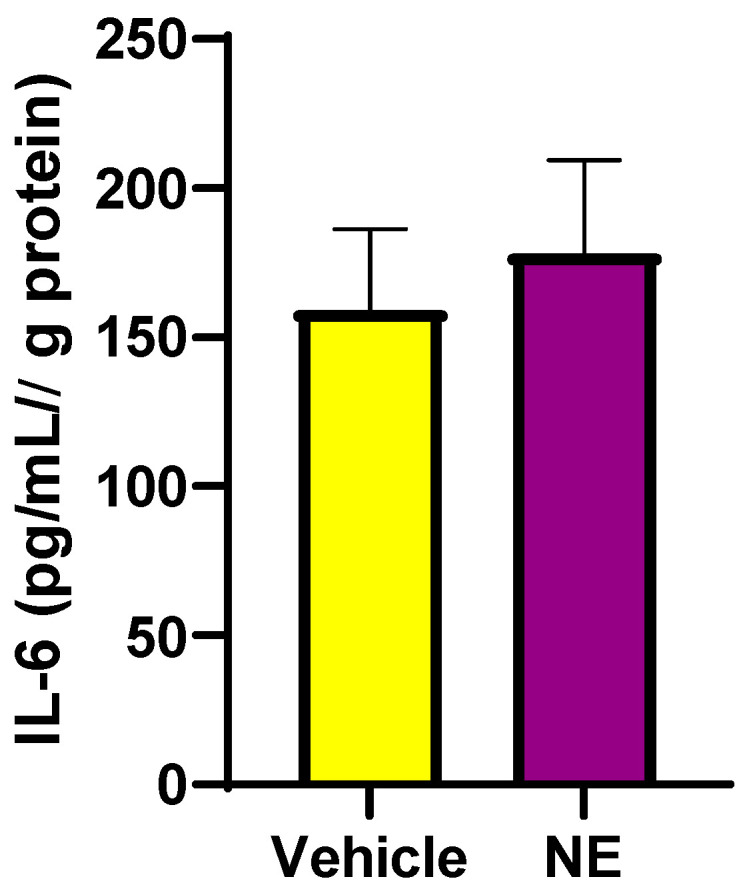
IL-6 protein levels are elevated following NE (10 μM) treatment of differentiated L6 cells. Whole-cell lysates were used for IL-6 determination. Data are represented as means ± SEM; n = 3/group.

Previously, it has been shown that the pro-inflammatory cytokine, IL-6, increases SNS activity [27], which may increase SGLT1 and SGLT2 levels in human proximal tubule cells [9,16]. In addition, IL-6 may directly increase SGLT1 levels [16]. We aimed to determine if NE increases IL-6 levels, which may possibly be upstream of elevated SGLT1 expression. In Figure 7, we demonstrate that NE does result in a trend for elevated IL-6 levels in differentiated skeletal muscle cells.

### 3.3. Determination of Significantly Increased SGLT1 in Skeletal Muscle of Neurogenically Hypertensive Mice (BPH/2J): Functional Implications on High Blood Pressure

#### 3.3.1. BPH/2J Hypertensive Mice Have Significantly Higher Blood Pressures Than BPN/3J Normotensive Mice

**Figure 8:**
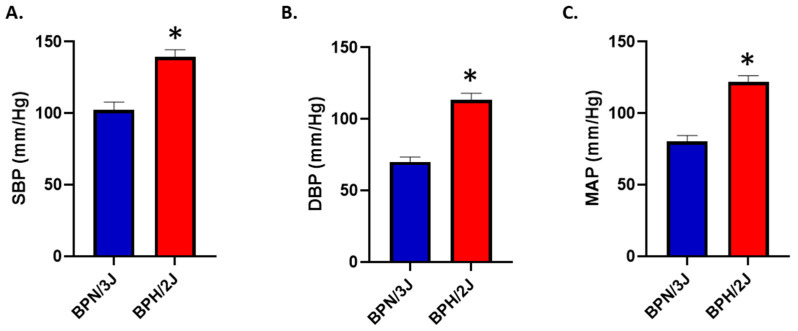
BPH/2J mice have significantly higher blood pressures than BPN/3J mice. (**A**) Systolic blood pressure (SBP), * *p* < 0.004; (**B**) diastolic blood pressure (DBP), * *p* < 0.001; (**C**) mean arterial pressure (MAP), * *p* < 0.001. n = 3–5 mice/group; statistical analysis was conducted using two-tailed Student’s *t*-tests. Blood pressure was determined in 13-week-old mice.

We have previously shown that neurogenically hypertensive (BPH/2J) mice display increased SNS activation, in numerous tissues such as the heart and the kidney [24]. We confirmed in an independent cohort of mice, that neurogenically hypertensive (BPH/2J) mice demonstrated increased systolic blood pressure (SBP; Figure 8A), diastolic blood pressure (DBP; Figure 8B), and mean arterial pressure (MAP; Figure 8C) compared to BPN/3J mice.

#### 3.3.2. Are SGLT1 Protein Levels Positively Regulated by Sympatho-Excitation in Skeletal Muscle In Vivo?

**Figure 9:**
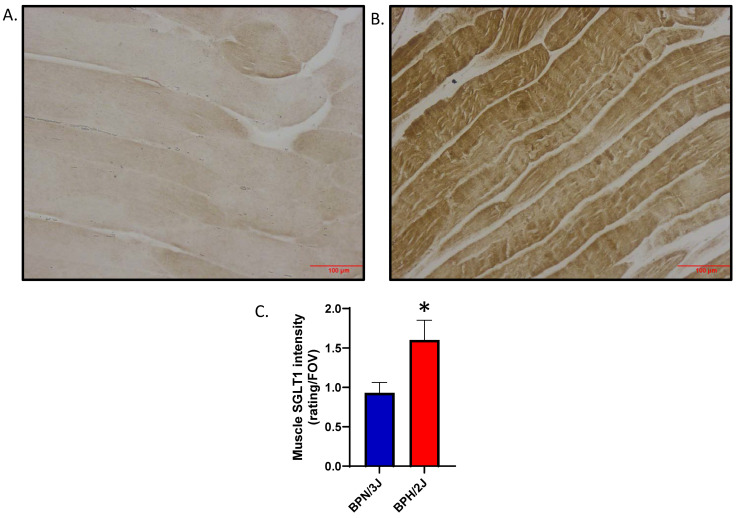
SGLT1 is significantly elevated in quadricep skeletal muscle of BPH/2J hypertensive mice compared to BPN/3J normotensive mice. (**A**) Representative image for BPN/3J mice; (**B**) Representative image for BPH/2J mice; (**C**) Quantitation of skeletal muscle SGLT1 intensity; 5–8 mice/group; data presented as means ± SEM; statistical analysis was conducted using two-tailed Student’s *t*-tests; * *p* = 0.024. Magnification in (**A**,**B**) is 200× and the scale bar is 100 μm.

We utilized our BPH/2J (sympathoexcitation mouse) and BPN/3J (normal SNS activity) mouse model to determine if the activation of the sympathetic nervous system increases SGLT1 protein levels. We demonstrated that SGLT1 is significantly increased in the quadricep skeletal muscle of our BPH/2J hypertensive mice compared to our normotensive BPN/3J mice (Figure 9). This suggests that when SGLT1 is increased in mice with a hyperactivated nervous system, such as BPH/2J mice, then the SNS positively regulates SGLT1 in vivo.

#### 3.3.3. Inhibition of SGLT1 and 2 with Sotagliflozin Lowers High Blood Pressure

**Figure 10:**
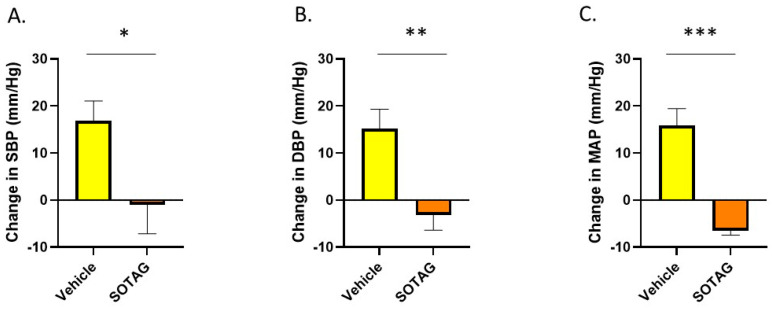
Sotagliflozin significantly lowers high blood pressure in BPH/2J mice after two weeks of therapy. (**A**) Systolic blood pressure (SBP); * *p* = 0.044, (**B**) diastolic blood pressure (DBP); ** *p* = 0.008, (**C**) mean arterial pressure (MAP); *** *p* = 0.006. n = 4–5 mice per group. Statistical analysis was conducted using two-tailed Student’s *t*-tests.

After determining that SGLT1 levels are increased in the skeletal muscle of the hypertensive mouse model, we assessed the effects of SGLT1/2 inhibition on blood pressure and confirmed that Sotagliflozin (SOTAG) administration resulted in a significant reduction in systolic blood pressure (SBP), diastolic blood pressure (DBP), and mean arterial pressure (MAP) following two weeks of treatment delivered via drinking water (Figure 10).

## 4. Discussion

Both hypertension and diabetes are chronic conditions which cumulatively affect over two billion people globally [28,29]. The levels of glucose in the blood and blood pressure are regulated by the sympathetic nervous system via the regulation of kidney function, peripheral vasculature tone, and metabolic control. The hyperactivation of the SNS has been associated with the development of DM and hypertension [30]. Similarly, the interplay between obesity and hypertension is evident. In particular, this is through the impact of obesity and perirenal adipose tissue on renal SNS activity [30], with downstream effects on the renin–angiotensin–aldosterone system and sodium retention [31].

It is clear that SGLT1 is expressed at a much higher level than SGLT2 in skeletal muscle. This aligns well with the tissue distribution of both proteins. The tissue distribution of SGLT1 is much greater than that of SGLT2. The SGLT2 protein is only expressed in the proximal tubule cells of the kidneys and the eyes, and we have now demonstrated for the first time its occurrence in skeletal muscle at a low but biologically functional level. In contrast, SGLT1 is more widely expressed throughout the body, including in organs such as the small intestine, the eyes, the kidneys, the heart, and skeletal muscle. This difference in expression between SGLT1 and SGLT2 could be due to the tighter transcriptional regulation of the *Sglt2* gene. Tissue-specific nucleosome occupancy has already been shown to play an important role in the differential regulation of *Sglt2* gene expression in various tissues [32]. This suggests that it is likely that, compared to the *Sglt1* gene in skeletal muscle, the DNA for the *Sglt2* gene in skeletal muscle may be more tightly wound around its supporting proteins to form chromatin, and this could affect the gene’s availability for transcription [32]. Additionally, the transcription factors for the *Sglt2* gene, such as C-Maf [33], may not be present in many tissues, which may explain the limited expression of SGLT2 in the body.

There is a vast amount of evidence highlighting that SGLT2i’s, Dapagliflozin, and Empagliflozin all promote metabolic benefits in skeletal muscle. Dapagliflozin promotes an improvement in fatty acid and ketone metabolism, as well as a reduction in glycolytic flux [34], while Empagliflozin improves insulin sensitivity [35] and enhances fatty acid oxidation [36] within skeletal muscle. Prior to our study, it may have been believed that these benefits were attributed to crosstalk between tissues. In our current study, we now show that SGLT2i is likely to be having a direct effect on skeletal muscle, as the target protein, SGLT2, is expressed at the protein level.

There are two main family members of the SGLT family, SGLT1 and SGLT2, with the former being less characterized in regard to its regulation. The SGLT2 protein has been widely studied for decades. Research has shown that in conditions of SNS hyperactivity, such as diabetes and hypertension, the function and expression of SGLT2 is perturbed. Rafiq et al. (2015) demonstrated that norepinephrine (NE), the major transmitter of the SNS, may increase *Sglt2* mRNA levels [37]. However, SGLT2 protein expression was not assessed. Regarding our own previous work, Matthews et al. (2017) was the first to show that SGLT2 protein expression can be positively regulated by the SNS in human proximal tubule cells (HK2) [9]. Our current study aimed to determine whether SNS activity also influences SGLT1 expression, particularly in the context of skeletal muscle. Our study demonstrated that neurogenically hypertensive mice had elevated levels of SGLT1 protein in the skeletal muscle. Whether elevated levels of NE were responsible for the increased SGLT1 in this tissue was unknown. We therefore conducted in vitro studies to assess whether NE may directly upregulate SGLT1 in skeletal muscle L6 cells. We did indeed show that NE promotes significantly increased SGLT1 expression in differentiated skeletal muscle L6 cells. This is not the first time that we have shown that NE can increase SGLT1. We have also demonstrated this to be the case in the context of human proximal tubule cells [16].

Various approaches are available to treat hypertension. Moxonidine is a pharmacotherapeutic antihypertensive drug that reduces peripheral sympathetic activity, thereby decreasing peripheral vascular resistance [38]. Renal Denervation (RDN) is an interventional procedure that utilizes radiofrequency or ultrasound ablation technologies to ablate renal nerves [39]. The resulting reduction in SNS activity has been associated with blood pressure lowering and reduction in renal glucose reabsorption [39]. It is plausible that the RDN-induced improvement in metabolic control may at least in part be mediated by SGLT downregulation caused by SNS inhibition. This demonstrates that the dysregulation of the SNS can be managed by appropriate medical treatment. Based on our results, it would be expected that the centrally acting Moxonidine would decrease both SGLT1 and SGLT2 levels in numerous organs.

Although our study demonstrates that SGLT1 and SGLT2 inhibitors may have direct effects on skeletal muscle, there are numerous benefits that SGLT inhibitors may have in tissues which do not express the target proteins. The heart is a classic example, as SGLT2is promote numerous beneficial actions in this tissue, even though cardiac tissue does not express SGLT2 [40]. SGLT1 inhibition with dual SGLT1/2is, such as Sotagliflozin, was previously thought to primarily delay SGLT1-mediated intestinal glucose absorption [40]. However, we now know that SGLT1/2is may also inhibit high levels of SGLT1 in skeletal muscle in vivo, and this may affect muscle function and glucose metabolism.

The limitations of our study include the following: (i) while the in vitro findings in L6 cells are valuable, they might not fully replicate in vivo conditions; (ii) the study uses specific concentrations and durations for NE, SGLT2i, and SGLT1/2i treatments, which may not cover the full spectrum of potential effects; and (iii) the study does not directly link SGLT1/2 expression changes to functional outcomes in muscle physiology or glucose metabolism.

As it is critical to translate our findings to humans, future studies should aim to determine whether SGLT1 and SGLT2 proteins are expressed in human skeletal muscle. It would be highly informative to determine if SGLT1 and SGLT2 are upregulated in patients with diabetes and/or hypertension in whom the SNS is hyperactivated. If SGLT1 and SGLT2 proteins are upregulated in these conditions, it would support our current pre-clinical findings and substantiate the use of the SGLT1/2i. Additionally, investigating different concentrations and treatment durations of NE and SGLT inhibitors could provide a more comprehensive understanding of their effects. Studies should explore the impact of altered SGLT1/2 expression on muscle function and glucose metabolism. As IL-6 is elevated in cell lysates with norepinephrine treatment, IL-6 should also be measured in cell culture supernatants. Lastly, given our study’s suggestion, research into the development and clinical testing of SGLT1-specific inhibitors could be promising for treating conditions with high SNS activity.

## 5. Conclusions

In this paper, our findings suggest that SNS activity upregulates the therapeutic target, SGLT1, in skeletal muscle, thereby potentially contributing to the well-described disturbance of cardiometabolic control. As clinical trial data suggest cardiorenal benefits with SGLT2 inhibitors (SGLT2i), future studies should aim to investigate the use of sole SGLT1 inhibitors (SGLT1i). This approach may provide a therapeutic strategy for conditions characterized by increased sympathetic nervous system (SNS) activity, such as hypertension, diabetes, and obesity.

## Data Availability

The data presented in this study are available on request from the authors.

## Supplementary Materials

The following supporting information can be downloaded at: https://www.mdpi.com/article/10.3390/biomedicines12071456/s1, Figure S1: Immunocytochemical detection of SGLT2 protein in differentiated L6 cells is not regulated by SGLT2i (EMPA) or SGLT1/2i (SOTAG).
