# Supplementary figures and images for: Sympathetic Activation Promotes Sodium Glucose Co-Transporter-1 Protein Expression in Rodent Skeletal Muscle
Source: Biomedicines. 2024 Jul 1;12(7):1456. doi: 10.3390/biomedicines12071456 (PMC11275186; doi:10.3390/biomedicines12071456)

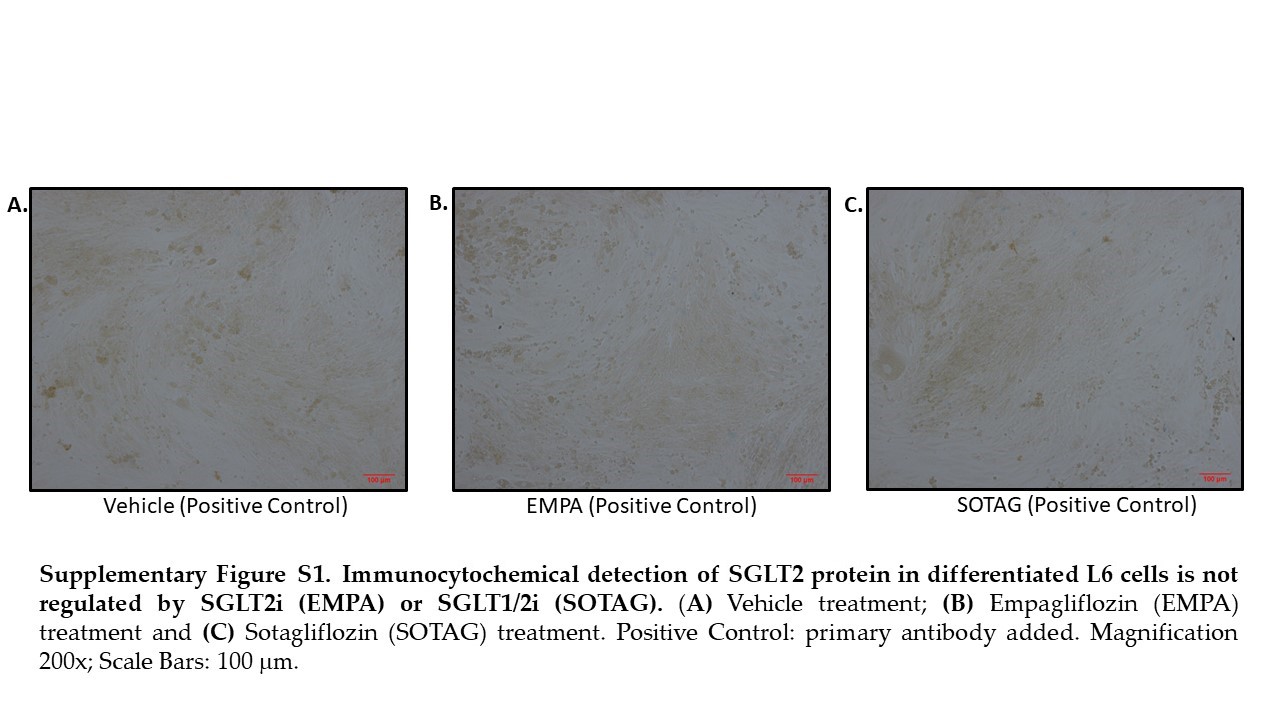

Supplement: Supplementary file 1 [file biomedicines-12-01456-s001.zip › biomedicines-3061001-supplementary.jpg]
